# Evidence for KISS-1 nuclear translocation and PI3K/AKT signaling in the ultrastructurally and morphometrically analyzed human endometriosis

**DOI:** 10.3389/fcell.2025.1625031

**Published:** 2026-01-06

**Authors:** Rasim Hamutoğlu, Celal Kaloğlu, Hüseyin Eray Bulut, Çağlar Yıldız

**Affiliations:** 1 Department of Histology and Embryology, Faculty of Medicine, Sivas-Cumhuriyet University, Sivas, Türkiye; 2 Assisted Reproduction Technology (ART) Center, Sivas-Cumhuriyet University, Sivas, Türkiye; 3 Department of Obstetrics and Gynaecology, Faculty of Medicine, Sivas-Cumhuriyet University, Sivas, Türkiye

**Keywords:** autophagy, endometriosis, intranuclear inclusions, JB-4 embedding technique, kisspeptin, mitochondrial degeneration, PI3K/Akt signaling pathway, stromal remodelling

## Abstract

**Background:**

Endometriosis is a common estrogen-dependent disease marked by ectopic endometrial growth. Although the PI3K/AKT and kisspeptin pathways are known to regulate endometrial homeostasis, their interplay in disease progression remains unclear. This study investigated the relationship between nuclear Kisspeptin (KiSS-1) localization and PI3K/AKT pathway activity in endometriotic tissues, focusing on stage-specific cellular alterations.

**Methods:**

In this prospective study, control, eutopic and ectopic endometrial biopsies were collected from 27 women (18 controls, 9 with ovarian endometriosis). Histopathological assessments were performed using JB4 embedding, immunofluorescence, and transmission electron microscopy. Morphometric analyses were used to quantify structural alterations.

**Results:**

In both eutopic and ectopic endometrium from patients with endometriosis, PI3K and AKT expression levels were significantly increased, whereas KiSS-1 expression was reduced and showed nuclear localization in a subset of cells. TEM analysis revealed features consistent with cellular stress, including autophagy-related vesicles, mitochondrial structural disruption, and alterations in nuclear architecture. Morphometric evaluation demonstrated a fibrotic remodeling in ectopic tissue. Specifically, glandular volume decreased, while stromal matrix content increased (p < 0.05).

**Conclusion:**

These findings suggest a mechanistic link between PI3K/AKT signaling and nuclear KiSS-1 translocation as an adaptive response to chronic hypoxia and inflammation in endometrial cells. This interaction may regulate survival, proliferation, and fibrotic remodeling processes characteristic of endometriosis. This integrated ultrastructural and molecular analysis provides novel insights into the pathophysiological role of nuclear KiSS-1 and its potential as a diagnostic and therapeutic target in endometriosis.

## 1 Introduction

The human endometrium undergoes cyclical changes under the influence of estrogen and progesterone, which regulate gene expression and cell signaling through membrane receptors and intracellular pathways such as mitogen-activated protein kinase/extracellular regulated protein kinase (MAPK/ERK) and phosphatidylinositol 3-kinase/protein kinase B (PI3K/AKT) (Marquardt et al., 2019). Disruption of endometrial homeostasis can lead to progesterone resistance and abnormal estrogen-dependent proliferation, both implicated in the pathogenesis of endometriosis and endometrial carcinoma (Anupa et al., 2019).

Endometriosis is an estrogen-dependent chronic inflammatory condition closely linked to severe pelvic pain, progressive dysmenorrhea, dyspareunia, and subfertility (Brown and Farquhar, 2014; Bedaiwy et al., 2017; Alimi et al., 2018; Coxon et al., 2018; Grogan et al., 2018; Bulun et al., 2019). It is defined as the presence and growth of endometrium-like tissue outside the uterine cavity and myometrium (Ma et al., 2021). Its true prevalence is likely underreported, and while theories such as menstrual blood reflux and endometrial implantation are widely accepted, the exact etiology remains unclear (Baranov et al., 2018; Shafrir et al., 2018; Agarwal et al., 2019; Zeppernick et al., 2020; Zhou et al., 2021). Recent studies suggest that endometriosis may serve as an early marker for ovarian endometrioid carcinoma (Zhao et al., 2018; Gaia-Oltean et al., 2021). In these patients, retrograde cell apoptosis was greatly diminished in endometriosis patients and the sensitivity of ectopic cells to apoptosis was decreased (Bilotas et al., 2010; Wang et al., 2015), and ectopic cells reduced sensitivity to apoptosis, contributing to their increased survival and implantation potential (Chung et al., 2002; Zhou et al., 2021).

Kisspeptins (formerly metastins), were initially identified as metastatic tumor suppressor peptides, but are now recognised for their crucial role in reproductive regulation (Lee et al., 1996; Pinilla et al., 2012). Emerging evidence indicates that KiSS-1 may have additional functions beyond its classical cytoplasmic role, including nuclear translocation and transcriptional modulation under specific cellular conditions (Mueller et al., 2011). Such regulatory mechanisms have been linked to stress-responsive pathways, as illustrated by nuclear GDF15 enhancing KISS-1 transcription via Sp1-dependent promoter activation (Zhou et al., 2021). Given the central role of PI3K/AKT signalling in cellular survival, metabolism, and stress adaptation, alterations in this pathway may influence KiSS-1 expression or localization dynamics. Notably, previous studies reported inconsistent KiSS-1 expression in endometriotic lesions (Alian et al., 2015; Timologou et al., 2016; Blasco et al., 2020) highlightining the need to clarify whether PI3K/AKT activity contributes to KiSS-1 deregulation.

The PI3K/AKT pathway plays a central role in cell survival, proliferation, apoptosis, and stress adaptation, and its dysregulation is a hallmark of endometriosis-related ovarian neoplasms (ERONs) (Che et al., 2015; Hemmings and Restuccia, 2015). Alterations such as PTEN loss, AKT hyperactivation, and PI3KCA mutations have been observed in both eutopic and ectopic endometrial tissues, promoting proliferation, invasion, epithelial–mesenchymal transition, and resistance to apoptosis (Mafra et al., 2014; Kandoth et al., 2013; Kiesel and Sourouni, 2019; Madanes et al., 2020). Mechanistic studies using *in vitro* and *in vivo* ERON models demonstrate that AKT activation, particularly with ARID1A loss and c-Myc overexpression, can drive malignant transformation of endometriotic epithelial cells (Matson et al., 2017; Shi et al., 2019; Beddows et al., 2024; Sohel et al., 2025). These findings provide a mechanistic rationale for investigating the interplay between PI3K/AKT signaling and KiSS-1 regulation in endometriosis and its potential progression toward malignancy (Kim et al., 2014; Matsuzaki et al., 2018).

The objective of this study was to investigate the relationship between KiSS-1 protein expression and PI3K/AKT pathway activity in endometriotic tissues, focusing on stage-specific localization patterns. By integrating ultrastructural and morphometric analyses, we sought to reveal the cellular and subcellular alterations that may reflect endometriosis progression and to establish robust morphological and quantitative reference points to enhance understanding of disease mechanisms and support improved diagnostic and therapeutic strategies.

## 2 Materials and methods

### 2.1 Ethical approval and patient recruitment

The study was approved by the Sivas Cumhuriyet University Clinical Research Ethics Committee (document number 2023-09/12). It was conducted in accordance with the Declaration of Helsinki (2013 revision). Written informed consent was obtained from all participants.

**FIGURE 1:**
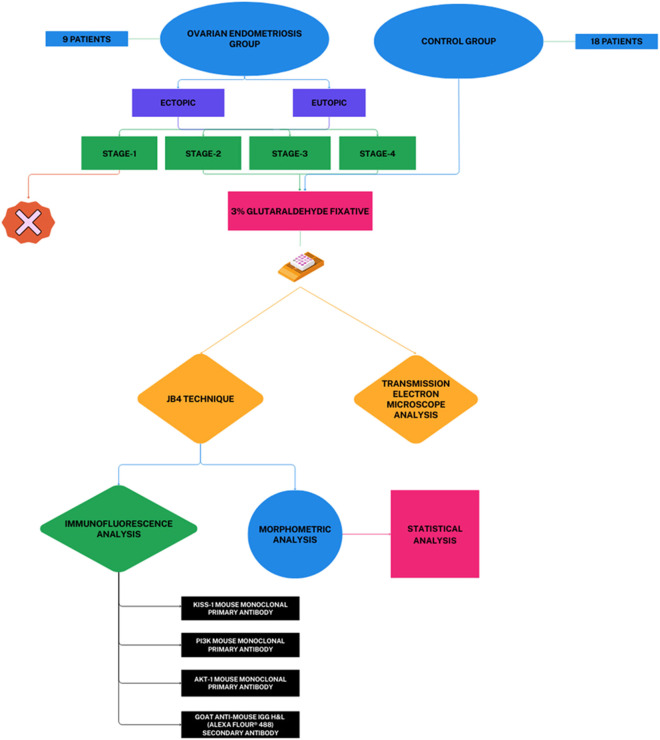
A schematic representation of the conducted study. Diagram outlines experimental workflow, including tissue collection, JB-4 embedding, immunofluorescence staining, ultrastructural TEM analysis, and morphometric quantifications.

Patients aged 18–50 years with regular menstrual cycles and endometriotic lesions >30 mm were included. Exclusion criteria were hormonal therapy within the last 3–6 months or endocrine/autoimmune disorders. Five control group biopsies were insufficient for evaluation, and one Stage-1 endometriosis patient was excluded, resulting in 27 participants (18 controls and 9 with ovarian endometriosis). Staging followed the revised American Fertility Society (r-AFS) scoring system (Hudelist et al., 2021): Stage II (n = 2), Stage III (n = 6), Stage IV (n = 1) (Figure 1). Controls included patients with tubal or unexplained infertility without endometriosis or other pathology.

All biopsies were obtained during the proliferative phase to minimize hormonal variability, particularly avoiding elevated luteal-phase progesterone, which can influence PI3K/AKT signaling and KiSS-1 expression. This approach ensured a consistent hormonal background for reliable comparison between eutopic and ectopic tissues.

### 2.2 Tissue preparation with JB4 technique in light microscopy

#### 2.2.1 Fixation and embedding

Tissues were fixed in 3% glutaraldehyde (Merck, Germany) at room temperature for 48 h, dehydrated through graded ethanol, and embedded in glycol-metacrylate-based resin (Polysciences, Inc., United States). Polymerization was completed at 4 °C over 3 days.

#### 2.2.2 Sectioning and staining

Semi-thin sections (1–1.5 μm) were stained sequentially with Acid Fuchsin and Toluidine Blue (Sigma-Aldrich, United States). Imaging was performed using an Olympus BX51 light microscope (Tokyo, Japan).

### 2.3 Immunofluorescence staining protocol

#### 2.3.1 Antigen retrieval and blocking

1 µm serial JB4sections were washed in distilled water and antigen retrieval was performed using 10 mM citrate buffer (pH:6.0), in a microwave (550W, 5 min). Sections were washed in phosphate buffered saline (PBS)-Triton-X100 and were blocked in SuperBlock (Sky Tech Lab, United States) for 20 min to prevent nonspecific binding.

#### 2.3.2 Primary and secondary antibodies

Sections were incubated overnight with mouse monoclonal KiSS-1, PI3K and AKT-1 primary antibodies (Santa Cruz Biotechnology, United States) at a 1:50 dilution. After washing, goat anti-mouse IgG secondary antibody (Abcam, United States; 1:200) and DAPI nuclear staining (200 nm/mL, Invitrogen, United States) were applied. Positive controls (Prostate tissue) and negative controls (without primary antibody, Supplementary Figure S1) were included. Prostate tissue served as positive control based on the manufacturer’s datasheet, which indicates high KiSS-1 expression in this tissue, ensuring the specificity and functionality of the primary antibody. Tissues were embedded in JB-4 glycol methacrylate resin, which was selected because it preserves intracellular and nuclear structures with high resolution and enables semi-thin sectioning (1 µm) for detailed visualization of subcellular localization. Although JB-4 embedding is not commonly recommended for immunofluorescence or special stainings due to potential limitations in antigen accessibility, our most recent study on rat liver tissue demonstrated that JB-4 sections provided excellent visualization with special stains such as PAS and silver impregnation, which are often considered challenging in JB-4 (İnan et al., 2025). These findings confirmed that JB-4 embedding can yield reliable and detailed immunofluorescence staining, supporting its suitability for KiSS-1, PI3K, and AKT detection in the present study.

A semi-quantitative scoring system (0–4) was applied to evaluate immunofluorescence staining intensity and distribution. Staining was scored as 0 = no staining, 1 = weak, 2 = moderate, 3 = strong, and 4 = very strong. Three independent observers assessed each section, and the average score was calculated to represent the final expression level. This approach ensured reproducibility and minimized subjective bias in fluorescence quantification. Although objective quantification of nuclear and cytoplasmic fluorescence using ImageJ (FIJI) software is widely adopted in the literature, this step was not performed in the present study due to technical limitations. Instead, we relied on the semi-quantitative scoring system, which has been routinely applied in our laboratory and provides a reliable evaluation of protein localization and staining intensity.

This combined approach of JB-4 embedding and semi-quantitative scoring ensures high-resolution visualization and a reproducible assessment of KiSS-1, PI3K, and AKT expression and subcellular localization, while transparently acknowledging current methodological limitations regarding automated quantification.

### 2.4 Tissue preparation for transmission electron microscopy

#### 2.4.1 Fixation and embedding

Small tissue pieces (∼1 mm^3^) were initially fixed in 3% glutaraldehyde for 48 h. Following primary fixation, tissues were rinsed in 0.1 M phosphate buffer and post-fixed in 1% osmium tetroxide (OsO_4_) (Sigma-Aldrich, Germany) for 2 h. Dehydration was performed through a graded ethanol (30%, 50%, 70%, 90%, and 100%), ensuring gradual removal of water to prevent tissue distortion. Finally, tissues were infiltrated and embedded in epoxy resin (Epon, Sigma-Aldrich, United States) according to standard protocols, and polymerized at 60 °C for 48 h.

#### 2.4.2 Sectioning and staining

Following polymerization, tissue blocks were carefully trimmed to expose the embedded specimens, and excess resin was removed. Glass knives were prepared from special glass rods using an automated knifemaker. For each biopsy, two blocks were prepared to ensure sampling consistency. Semi-thin sections (∼0.5 μm) were cut using a Reichert ultramicrotome, stained with 0.05% toluidine blue (pH 4.4) in acetate buffer for 30 s at 60 °C, then air-dried and mounted. Once suitable areas were identified, blocks were re-trimmed as necessary. Silver-gold-colored ultrathin sections (∼70 nm) were cut with the same ultramicrotome and carefully collected onto 100-mesh (3.05 mm copper grids) coated with 4% collodion dissolved in amyl acetate. Sections were subsequently double stained with uranyl acetate (saturated in 70% ethanol) for 20 min in the dark, then with lead citrate for 5 min in a carbon dioxide (CO2)-free environment. Several sodium hydroxide pellets were placed in the lead citrate staining dish to absorb CO2.

#### 2.4.3 Imaging

10 electromicrographs per section (20 per individual) were randomly selected from the best technical areas. Images were acquired using a JEOL JEM-1400 TEM (JEOL, Japan) at 60 kV. A grating replica (2,160 lines/mm) was used for magnification calibration.

### 2.5 Morphometric analysis

Morphometric evaluations were performed on JB4-embedded sections at 40× magnification using a point-counting method with a systematic random sampling approach (Zhioua et al., 2012). Volume fractions of glands, stroma, and vessels were measured.

### 2.6 Statistical analysis

Prior to the study, a power analysis was conducted (α = 0.05 and β = 0.20), yielding a study power of 90.8%. Data analysis was performed using SPSS v.22.0 (IBM Corp., Armonk, NY). The distribution of the data was evaluated using the Kolmogorov–Smirnov test. In cases where the assumptions for parametric testing were not met, the Mann–Whitney U test was used for comparisons between two groups, and the Kruskal–Wallis test was used for comparisons involving three or more groups. When parametric assumptions were met, the independent samples t-test was applied to assess differences between group means. In all analyses, the level of significance was set at p < 0.05 (Aksakoğlu, 2001).

## 3 Results

### 3.1 General description of biopsies

Biopsies were obtained from the ectopic and eutopic endometrium of women with endometriosis, who most mainly presented with abnormal uterine bleeding (50%), dysmenorrhea (35%), chronic pelvic pain (1%), and dyspareunia (14%). Control biopsies were also collected. All samples were histologically confirmed to be in the proliferative phase of the menstrual cycle. Demographic and clinical data of endometriosis patients were summarized in Table 1.

**TABLE 1:** Distribution of histopathological features of the cases.

Patient	Stage	Age	Complaint	Associated lesion(s)	HLM	Stroma/Gland	Fibrosis	Atypia
1	3	41	Abnormal uterine bleeding	IC	+	S	-	+
2	2	29	Dysmenorrhea	CLH	-	S	+	-
3	4	43	Dyspareunia	FC, CLH	-	SG	+	-
4	3	46	Dyspareunia	FC, CLH, CA	+	S	+	+
5	3	50	Abnormal uterine bleeding	FC, CA	+	S	+	-
6	3	49	Abnormal uterine bleeding	PTC, CA	+	SG	+	+
7	2	35	Chronic pelvic pain	FC, PTC, CA	+	S	+	+
8	3	43	Abnormal uterine bleeding	FC, CLH	+	S	+	-
9	3	44	Dysmenorrhea	FC, CLH	+	S	+	+

IC: inclusion cysts, CLH: corpus luteum hemorrhagicum, FC: follicular cysts, CA: corpus albicans, PTC: paratubal cysts, HLM: Hemosiderin-laden macrophages.

### 3.2 Light microscopy findings using the JB4 technique

#### 3.2.1 Control group

**FIGURE 2:**
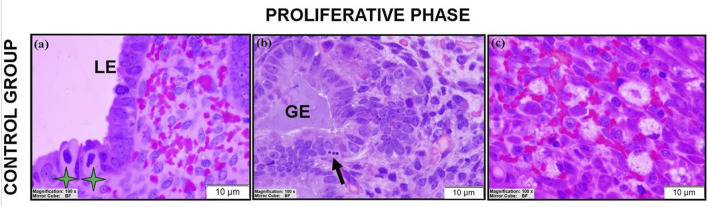
Morphological findings of control endometrial sections obtained with the JB4 technique. Representative images show the luminal epithelium (LE) and glandular epithelium (GE), with atypical cells (green asterisks) and epithelioid cells (thick black arrow), highlighting normal proliferative phase architecture. Scale bars = 10 μm.

Histological evaluation revealed three luminal epithelial types: simple cuboidal, simple columnar, and pseudostratified columnar epithelium (Figure 2a; see also Supplementary Figure S2a,b,d). Simple glandular hyperplasia was frequently observed (Figure 2b; Supplementary Figure S2c,e,f). Occasional atypical cells with increased cytoplasm-to-nucleus (Figure 2a; Supplementary Figure S2d) and foam cells (Figure 2c) were noted. Mitotic figures were identified in both the luminal epithelium and stroma (Supplementary Figure S2b,f), along with epithelioid cells (Figure 2b).

#### 3.2.2 Ectopic endometriosis group

**FIGURE 3:**
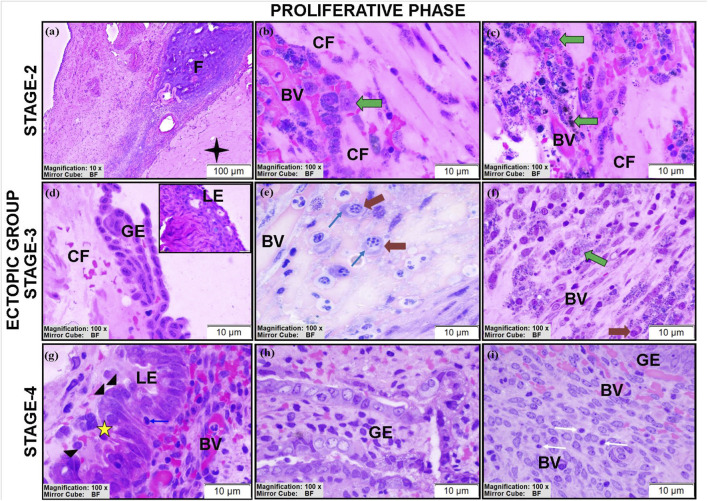
Morphological findings of ectopic endometrial sections obtained with the JB4 technique. Images illustrate LE, GE, blood vessels (BV), fibrotic areas (F), collagen fibers (CF), corpus albicans (black asterisk), hemosiderin-laden macrophages (green arrows), plasma cells (brown arrows), Russell bodies (light blue arrows), intraepithelial leukocytes (blue arrows), supranuclear vacuolization (yellow asterisks), and spindle-like cells (black arrowheads), indicating tissue remodeling and inflammation in ectopic lesions. Scale bars: A = 100 μm, B-I = 10 µm.

Ectopic tissues, demonstrated endometrial structures infiltrating the ovarian wall and stroma (Figure 3a), accompanied by simple or complex hyperplasia (Figures 3d,h,i). In stage II lesions, ectopic glands adhered to ovarian tissue, while endometriotic cyst with dense fibrosis was often located adjacent to the corpus albicans (Figure 3a).

A wide range of cellular morphology within luminal and glandular epithelia and stromal regions, including prominent nucleoli, euchromatic or hyperchromatic nuclei, supranuclear vacuolizations, spindle-like cells, inflammatory cells, intraepithelial leukocytes, and gland-in-gland patterns, was noted, particularly in stage IV lesions (Figures 3g–i).

The stroma exhibited decreased cellularity and increased collagen density (Figures 3b–d). Hemosiderin-laden macrophages (HLMs) were abundant in stages II and III (Figures 3b,c,f), but absent in stage IV. Plasma cells and Russell bodies were also identified (Figures 3e,f).

#### 3.2.3 Eutopic endometrium in endometriosis patients

**FIGURE 4:**
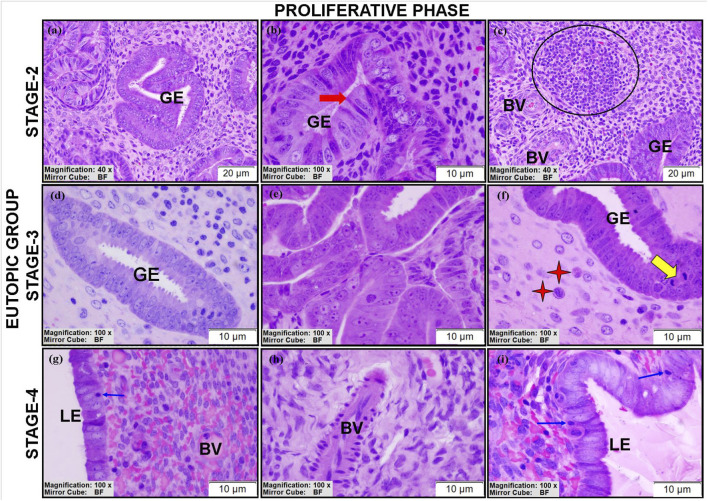
Morphological findings of eutopic endometrial sections obtained with the JB4 technique. Representative images show LE, GE, BV, mitotic figures (yellow arrows), lymphoid aggregations (black circular area), tubal metaplastic cells (red arrow), mast cells (red asterisks), and intraepithelial leukocytes (blue arrows), highlighting immune cell infiltration and early metaplastic changes. Scale bars: A,C = 20 μm, B,D-I = 10 µm.

The eutopic endometrium showed more complex alterations than the control group. Stage II eutopic tissues showed complex atypical hyperplasia with back-to-back glandular formations and stromal compression (Figure 4a). Glandular epithelial cells exhibited heterogeneity, including prominent nucleoli, apical secretory blebs, and tubal metaplastic (peg) cells (Figures 4b,c).

Stage III eutopic tissues predominantly showed simple hyperplasia (Figures 4d,f), although one case exhibited complex atypical hyperplasia (Figure 4e). Mitotic figures and intraepithelial leukocytes were noted in the glands (Figures 4f,g,i). Mast cells (Figure 4f) were also present.

The eutopic stroma remained high cellular (Figures 4a–i) and contained lymphoid aggregates with pyknotic nuclei (Figure 4c). Hyperchromatic stromal cells surrounded some glands (Figure 4d). Thick-(Figure 4h) and thin-walled blood vessels (Figures 4c,g) were noted across stages, although glandular structures were absent in stage IV eutopic tissues.

### 3.3 Immunofluorescence findings using the JB4 technique

#### 3.3.1 KiSS-1 protein expression

KiSS-1 was detected in all groups with variable intensity and localization (Table 2).

**FIGURE 5:**
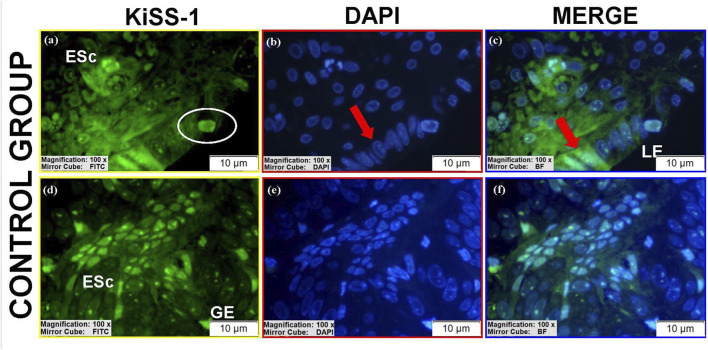
Immunofluorescence localizations of KiSS-1 protein in endometrial tissues of control group. GE, LE, and endometrial stromal cells (ESc) are shown. Atypical cells (white circular areas) and spindle-shaped cells (red arrows) illustrate subcellular distribution of KiSS-1. Scale bars = 10 μm.

**FIGURE 6:**
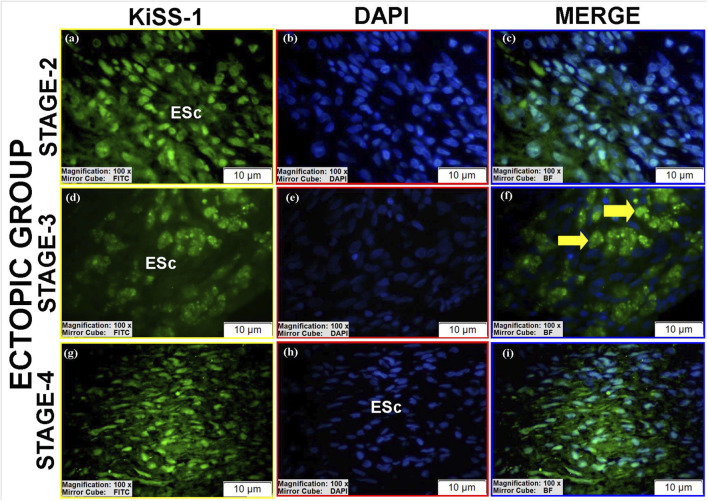
Immunofluorescence localizations of KiSS-1 protein in endometrial tissues of ectopic group. ESc and hemosiderin-laden macrophages (yellow arrow) are indicated, highlighting altered KiSS-1 expression patterns in ectopic lesions. Scale bars = 10 µm.

**FIGURE 7:**
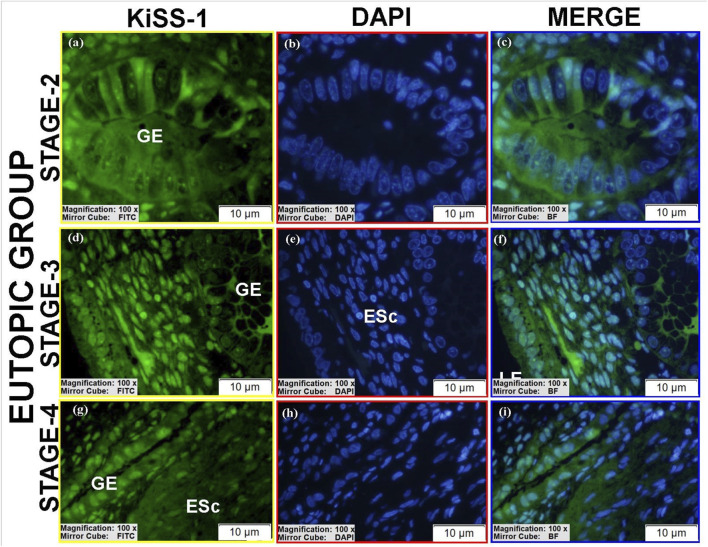
Immunofluorescence localizations of KiSS-1 protein in endometrial tissues of eutopic group. GE, LE, and ESc are shown, illustrating differential nuclear and cytoplasmic KiSS-1 localization relative to control tissues. Scale bars = 10 µm.

- Luminal epithelium: Low perinuclear expression was observed in controls and stage II eutopic tissues (Figures 5a–c; Supplementary Figure S3a–f), whereas stage III eutopic tissues showed prominent nuclear expression (Figures 7d–f). Luminal epithelium was not reliably identifiable in stage IV eutopic and all ectopic tissues.
- Glandular epithelium: Stage III eutopic samples showed strong cytomembrane staining (Figures 7d–i; see also Supplementary Figure S3g–i). Stage II eutopic glands had heterogeneous patterns, including spindle-shaped cells with intense nuclear and cytoplasmic positivity (Figures 7a–c).
- Stroma: Ectopic tissues showed high nuclear KiSS-1 expression in stages II and IV (Figures 6a–c,g–i), and predominantly cytoplasmic staining in stage III (Figures 6d–f). Eutopic stroma exhibited intense perinuclear and nucleolar signals (Supplementary Figure S3g–i; Figures 7d–f). Control stroma showed high nuclear expression, while decidua-like cells lacked staining (Figures 5d–f). Vascular endothelial expression was uniformly low across groups.

**TABLE 2:** KiSS-1 immunolocalization in ectopic and eutopic endometrium of patients with endometriosis and endometrial tissues of control patients without endometriosis.

Tissue type/Tissue regions		KiSS-1						
		HLM	Luminal epithelium	Glandular epithelium	Cells within the stroma			Endothelium
					Cytoplasm	Nucleus	Perinuclear area	
*Stage-2*	*Ectopic*	+++	*	*	-	+++	+	+++
	*Eutopic*	*	+ (pn)	++	-	+++	+++	+/++
*Stage-3*	*Ectopic*	++	*	*	++	+	-	−/+
	*Eutopic*	*	++/+++ (n)	++/+++ (cm)	-	+++	+++	−/+
*Stage-4*	*Ectopic*	*	*	+/++	-	+++	-	+
	*Eutopic*	*	*	+ (n)	-	+++	-	+
*Control group*		*	+	+/++	+	++++	+++	+/++

++++; Very strong expression, +++; Strong expression, ++; Moderate expression, +; Weak expression, -; no expression, * The indicated structure was not observed. n→ nuclear area, pn→ perinuclear area, cm→ cell membrane.

#### 3.3.2 PI3K protein expression

PI3K expression varied across groups (Table 3).

**FIGURE 8:**
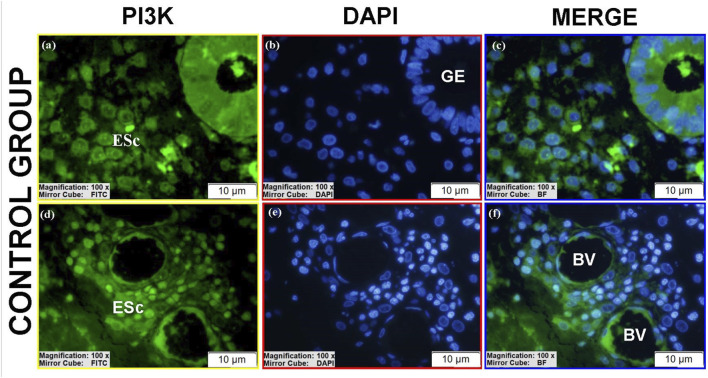
Immunofluorescence localizations of PI3K protein in endometrial tissues of control group patients. GE, BV, and ESc are shown, highlighting baseline cytoplasmic PI3K expression in normal endometrium. Scale bars = 10 µm.

**FIGURE 9:**
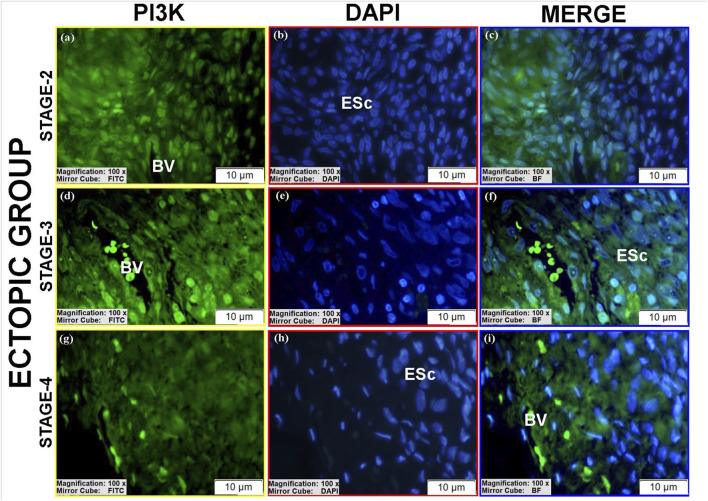
Immunofluorescence localizations of PI3K protein in endometrial tissues of ectopic group patients. BV and ESc are indicated, illustrating increased nuclear and perinuclear PI3K in stromal cells, with vascular staining unchanged. Scale bars = 10 µm.

**FIGURE 10:**
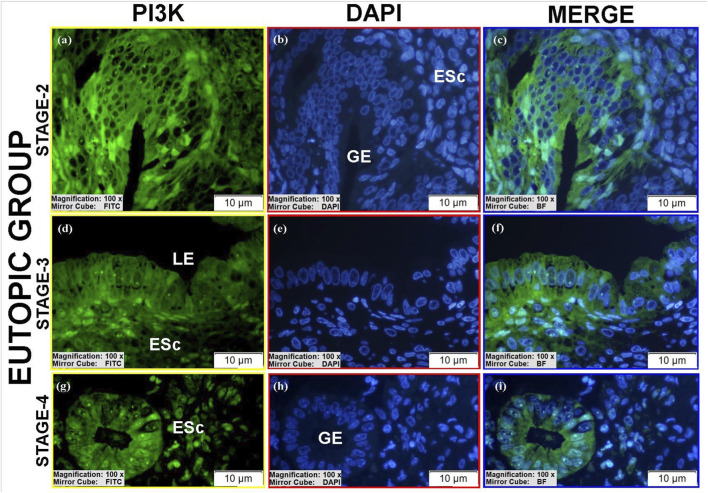
Immunofluorescence localizations of PI3K protein in endometrial tissues of eutopic group patients. GE: Gland epithelium, LE: Luminal epithelium, ESc: Endometrial stromal cells. Scale bars = 10 µm.

- Luminal epithelium: Moderate to high PI3K immunoreactivity was present in stages II-III ectopic and stage III eutopic tissues (Figures 10d–f; see Supplementary Figure S4a–c).
- Glandular epithelium: Controls showed moderate cytoplasmic staining (Figures 8a–c), whereas eutopic glands displayed markedly higher PI3K levels, particularly in spindle-shaped cells with strong nuclear and cytoplasmic positivity (Figures 10a–c,g–i; Supplementary Figure S4d–f).
- Stroma: Ectopic and eutopic tissues showed reduced cytoplasmic staining but increased nuclear/perinuclear localization (Figures 8a–f; Figures 9a–i; Figures 10a–i). Stage III ectopic tissues also displayed increased vascular endothelial staining (Figures 9d–f), but overall vascular staining did not differ significantly between groups (Figures 8d–f). Stromal cells with pyknotic nuclei exhibited particularly high nuclear PI3K expression (Figures 8d–f). Stage IV tissues showed reduced PI3K expression overall. The reduced glandular structures observed in ectopic lesions may be attributed to chronic inflammatory stress, stromal fibrosis, and extracellular matrix remodeling, which can disrupt normal glandular architecture and potentially influence PI3K signaling patterns.

**TABLE 3:** PI3K immunolocalization in ectopic and eutopic endometrium of patients with endometriosis and endometrial tissues of control patients without endometriosis.

Tissue type/Tissue regions		PI3K						
		HLM	Luminal epithelium	Glandular epithelium	Cells within the stroma			Endothelium
					Cytoplasm	Nucleus	Perinuclear area	
*Stage-2*	*Ectopic*	*	+++	*	-	+++	+++	^+^
	*Eutopic*	*	++	++/+++	-	+++	+	+/++
*Stage-3*	*Ectopic*	+++	++/+++	*	+	+++	+++	++/+++
	*Eutopic*	*	++/+++	++/+++	-	+++	+++	++/+++
*Stage-4*	*Ectopic*	*	*	*	+	+/++	+	−/+
	*Eutopic*	*	*	++/+++	-	+/++	+++	+ (pn)
*Control group*			++	++	+++	+	-	+++

++++; Very strong expression, +++; Strong expression, ++; Moderate expression, +; Weak expression, -; no expression, * The indicated structure was not observed. pn→ perinuclear area.

#### 3.3.3 AKT-1 protein expression

AKT-1 was detected in all groups (Table 4).

**FIGURE 11:**
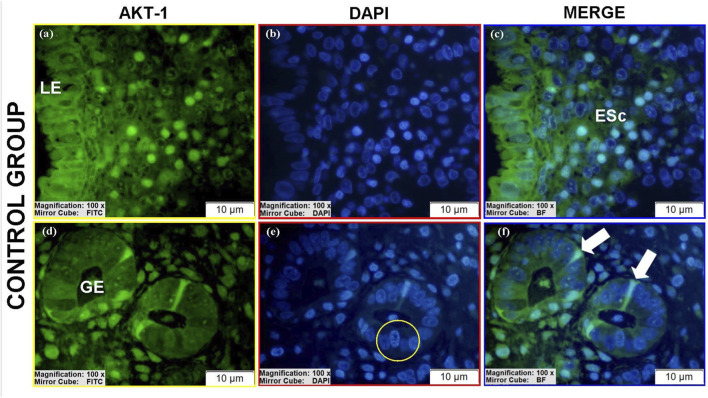
Immunofluorescence localizations of AKT-1 protein in endometrial tissues of control group patients. GE: Gland epithelium, LE: Luminal epithelium, ESc: Endometrial stromal cells, Cell in prophase stage (yellow circular area), Tubal metaplasia cells (white arrow). Scale bars = 10 µm.

**FIGURE 12:**
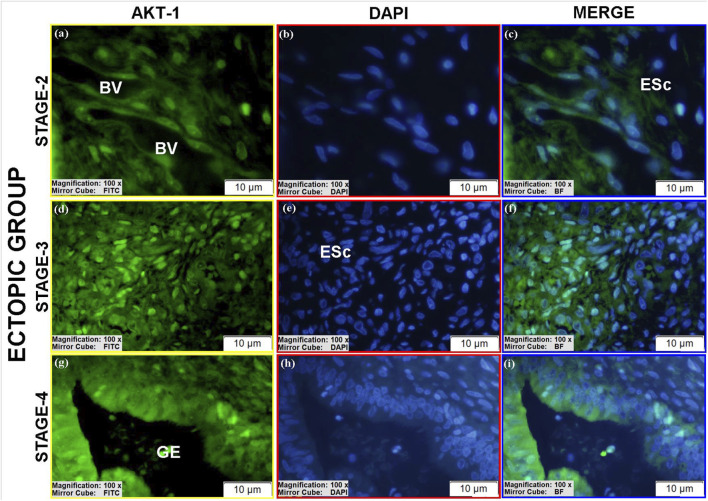
Immunofluorescence localizations of AKT-1 protein in endometrial tissues of ectopic group patients. GE: Gland epithelium, BV: Blood vessel, ESc: Endometrial stromal cells. Scale bars = 10 µm.

**FIGURE 13:**
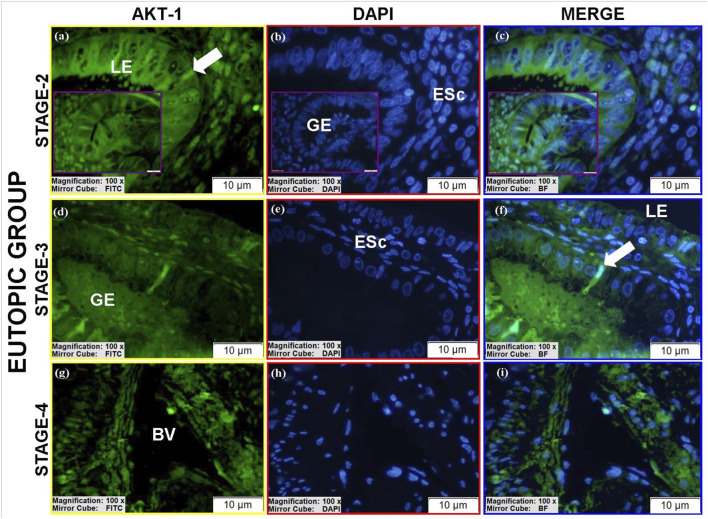
Immunofluorescence localizations of AKT-1 protein in endometrial tissues of eutopic group patients. GE: Gland epithelium, LE: Luminal epithelium, BV: Blood vessel, ESc: Endometrial stromal cells, Tubal metaplasia cells (white arrow). Scale bars = 10 µm.

- Luminal epithelium: Only stage II eutopic samples showed clearly identifiable luminal epithelium, with high cytoplasmic AKT-1 expression (Figures 13a–c), exceeding control levels (Figures 11a–c; see Supplementary Figure S5a–c).
- Glands: Ectopic and eutopic glandular epithelium exhibited strong AKT-1 expression, particularly in spindle-shaped cells (Figures 12g–i; Figures 13a–f). Controls showed minimal staining (Figures 11d–f).
- Stroma: AKT-1 localization shifted from predominantly cytoplasmic in controls to nuclear/perinuclear in ectopic and eutopic tissues (Figures 12a–f, 13a–f; Supplementary Figure S5d–l). Vascular endothelial staining remained low across groups (Figures 12a–c, 13g–i).

**TABLE 4:** AKT-1 immunolocalization in ectopic and eutopic endometrium of patients with endometriosis and endometrial tissues of control patients without endometriosis.

Tissue type/Tissue regions		AKT-1						
		HLM	Luminal epithelium	Glandular epithelium	Cells within the stroma			Endothelium
					Cytoplasm	Nucleus	Perinuclear area	
*Stage-2*	*Ectopic*	*	*	*	-	++	++	+ (n)
	*Eutopic*	*	++/+++	++/+++	-	++++	++	++
*Stage-3*	*Ectopic*	+++	*	*	-	+++	+	+ (pn)
	*Eutopic*	*	+/++	++/+++	-	++++	+	+
*Stage-4*	*Ectopic*	*	*	+++	-	−/+	−/+	+
	*Eutopic*	*	*	*	-	++	−/+	−/+
*Control group*			+/++	+	+++	++	++	+ (pn)

++++; Very strong expression, +++; Strong expression, ++; Moderate expression, +; Weak expression, -; no expression, * The indicated structure was not observed. n→ nuclear area, pn→ perinuclear area.

### 3.4 Transmission electron microscopy findings

#### 3.4.1 Control endometrium

**FIGURE 14:**
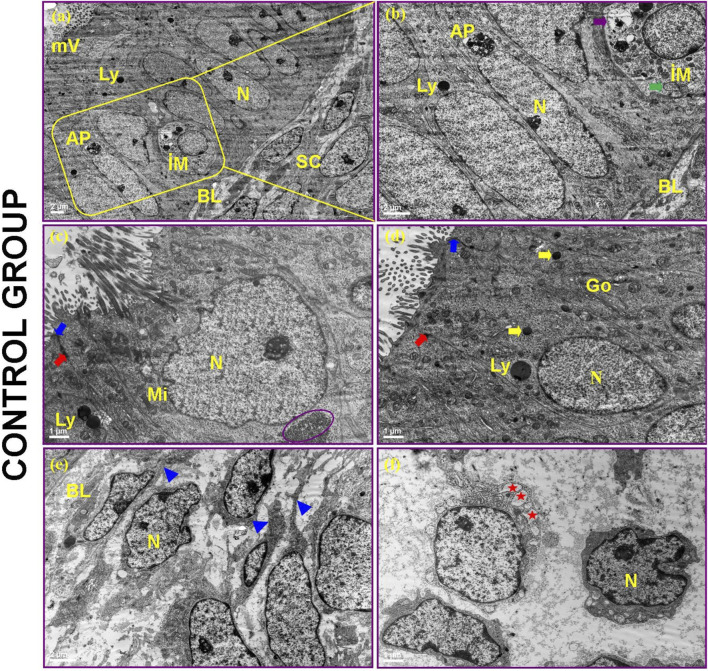
Electron microscopic view of control endometrium in the proliferative phase. Microvilli (mV), Nucleus (N), Lysosome (Ly), Autophagosome (AP), Intraepithelial macrophage (IM), Stromal cells (SC), Basal lamina (BL), Golgi apparatus (Go), Mitochondria (Mi), Small electron-dense intracytoplasmic granules (yellow arrow), Glycogen particles (purple circular area), Zonula occludens (blue arrow), Desmosomes (red arrow), Multi-vesicular bodies (green arrow), Heterolysosome (purple arrow), Cytoplasmic extensions (blue arrowheads), Vesicle (red asterisk).

TEM revealed ciliated and microvillous epithelial cells forming a simple columnar glandular epithelium (Figures 14a–d). Junctional complexes (tight junctions, desmosomes) and a continuous basal lamina were intact (Figures 14b–d). The stroma contained elongated fibroblast-like cells with euchromatic nuclei and minimal collagen deposition (Figures 14e,f). Intraepithelial macrophages with lysosomes and autophagosomes were observed (Figures 14a,b).

#### 3.4.2 Ectopic endometrium

**FIGURE 15:**
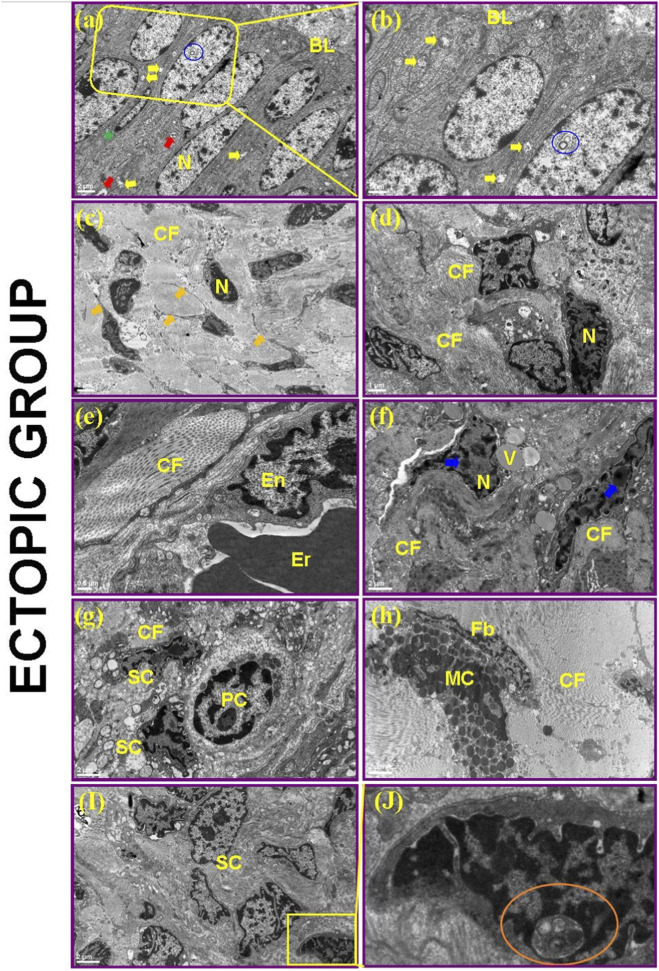
Electron microscopic view of ectopic endometrium in the proliferative phase. Nucleus (N), Mitochondria (Mi), Basal Lamina (BL), Collagen fibers (CF), Vesicles (V), Blood vessel endothelium (En), Atypical stromal cells (SC), Fibroblasts (Fb), Mast cells (MC), Plasma cells (PC), Erythrocyte (Er), Cellular structures showing tubal metaplasia (green asterisk), Intranuclear inclusion bodies (blue and orange circular areas), Heterolysosome (red arrow), Cytoplastic extensions (orange arrow), Stainings in the nucleus (blue arrow).

Ectopic lesions exhibited pseudostratified columnar epithelium with abundant ciliated and microvillous cells, as well as tubal metaplasia-like cells (Figures 15a,b). Numerous degenerated mitochondria and intranuclear inclusion bodies were observed in both epithelial and stromal cells (Figures 15a,b,i,j). These inclusions were distinguished from nuclear invaginations by their morphology: true inclusions appeared as discrete, membrane-bound structures entirely enclosed within the nuclear matrix, often containing electron-dense or granular material, whereas nuclear invaginations were continuous extensions of the nuclear envelope connecting to the cytoplasm. Multiple serial sections confirmed that inclusions were fully enclosed (Figures 15i,j). The stroma was fibrotic, with hyperchromatic, pleomorphic nuclei, nuclear indentations, dense collagen, and inflammatory infiltrates (Figures 15c–i).

#### 3.4.3 Eutopic endometrium

**FIGURE 16:**
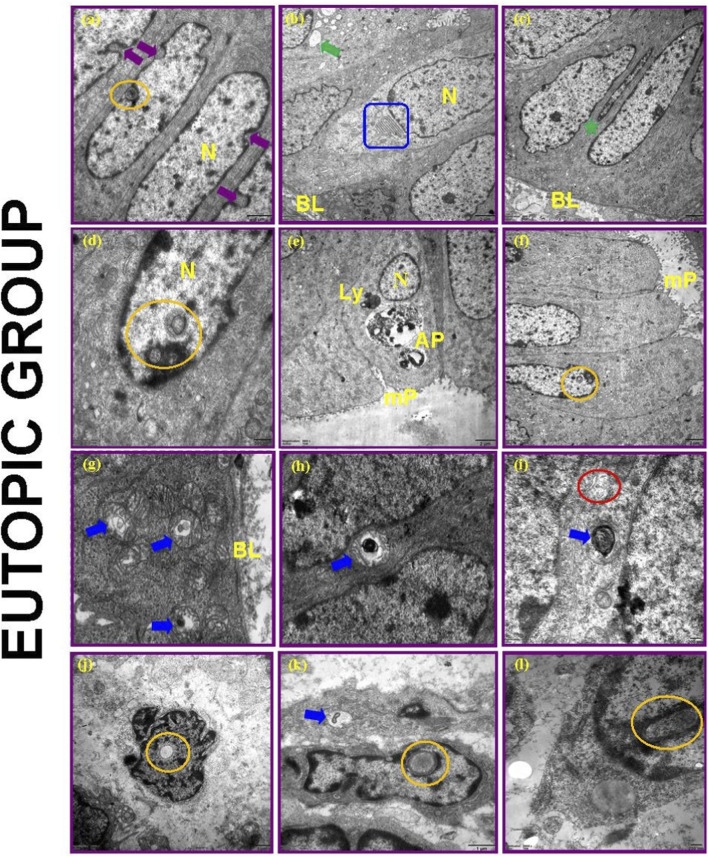
Electron microscopic view of eutopic endometrium in the proliferative phase. Nucleus (N), Basal lamina (BL), Rough endoplasmic reticulum (blue square), Lysosome (Ly), Giant autophagocytic lysosomes (AP), Microplica (mP), Cellular structures showing tubal metaplasia (green asterisk), Intranuclear inclusion bodies (yellow circular areas), Deep indentations (purple arrow), Mitochondrial degenerations (blue arrows), Widened appearance of intercellular lateral membranes (red circular area), Large vesicle (green arrow).

Eutopic tissue also showed pseudostratified glandular epithelium with tubal metaplasia and prominent microplicae (Figures 16b,c,e,f). Mitochondrial degeneration (Figures 16g–i,k) and intranuclear inclusions (Figures 16a,d,f,j–l) similar to those in ectopic tissues were present. Intranuclear inclusions were confirmed as discrete, membrane-bound structures fully separated from the cytoplasm, distinguishing them from nuclear invaginations. The stroma exhibited more regular nuclear morphology with evident edema (Figures 16j–l). Giant autophagocytic lysosomes (Figure 16e) and metallic cytoplasmic deposits (Figures 16g–i) were also noted.

### 3.5 Morphometry

#### 3.5.1 Total area volume fraction

**FIGURE 17:**
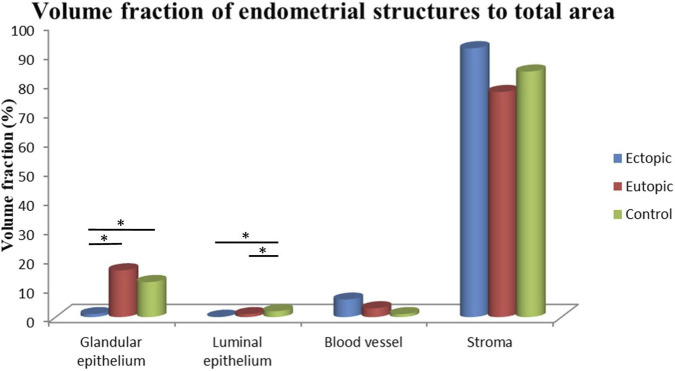
Total area volume fractions (%) of endometrial structures across the study groups. Data are presented as mean ± SD. ^*^p < 0.05 compared to control group, highlighting structural alterations in ectopic and eutopic tissues.

- Glandular Epithelium: Eutopic endometrium had highest volume fraction (16%), significantly exceeding ectopic tissue (lowest values; p < 0.05). Controls measured 12%. Pairwise comparisons revealed a significant difference between the ectopic and eutopic groups, as well as between the ectopic and control groups (p < 0.05), whereas no significant difference was found between the eutopic and control groups (p > 0.05) (Figure 17). Furthermore, when comparing the cells and lumen of the glandular epithelium, it was observed that the volume fraction in both the eutopic and control groups was approximately the same (87%). No statistically significant difference was found between these two groups (p > 0.05).
- Luminal Epithelium: Controls showed the highest volume fraction (2%), whereas ectopic tissues showed lowest (0.1%) (p < 0.05). Significant differences were found between the ectopic and control groups and between the eutopic and control groups (p < 0.05), but no significant difference was observed between the ectopic and eutopic groups (p > 0.05) (Figure 17).
- Blood Vessels: The ectopic endometrium exhibited the highest volume fraction for blood vessels at 6%, whereas the control group had the lowest volume fraction at 1%. Despite the apparent difference in volume fractions, no statistically significant differences were observed between the groups (p > 0.05) (Figure 17).
- Stroma: The ectopic group had the highest volume fraction for the stroma at 92%, while the eutopic endometrium showed the lowest at 77%. There were no significant differences between the groups in terms of stromal volume fraction (p > 0.05) (Figure 17).

#### 3.5.2 Cell/matrix ratio

**FIGURE 18:**
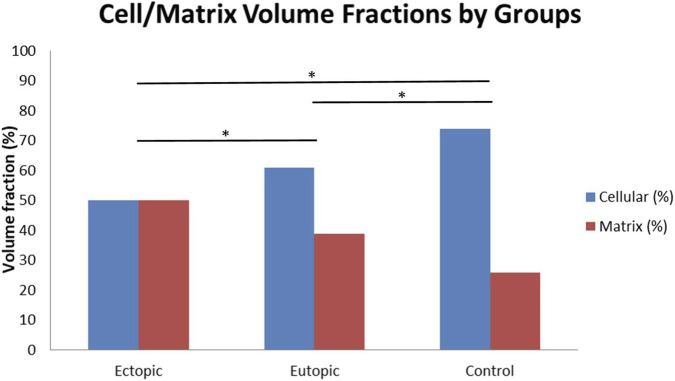
Cell-to-matrix volume fraction ratios (%) in the stromal tissue across the study groups. ^*^p < 0.05 compared to control group, indicating altered stromal composition in endometriosis.

The volume fraction ratios of the cellular and matrix components within the stromal tissue were analyzed among the study groups. The stromal cell volume fractions were measured as 50% in the ectopic group, 61% in the eutopic group, and 74% in the control group. Conversely, the matrix volume fractions were recorded as 50%, 39%, and 26%, respectively (Figure 18).

These findings indicate a progressive decrease in cellular components and a concomitant increase in matrix components within the stroma of the ectopic endometrium, suggesting enhanced fibrotic activity and stromal remodeling in ectopic lesions. Pairwise comparisons revealed statistically significant differences in cell/matrix ratios among the groups (ectopic vs. eutopic, ectopic vs. control, and eutopic vs. control; p < 0.05).

## 4 Discussion

Endometriosis is a benign yet increasingly prevalent condition, likely reflecting advances in diagnostic methods. Traditional anatomical definitions fall short of capturing the full spectrum of its natural history, chronicity, and clinical heterogeneity. This gap underscores the need for a patient-centered conceptualization, which encompasses the cellular, molecular, and systemic aspects of disease progression (Bulun et al., 2019). Our study identified significant morphological and morphometric differences between ectopic and eutopic endometrial tissues. KiSS-1 showed apparent nuclear localization in several samples; however, dedicated methods required to confirm a true cytoplasmic-to-nuclear translocation. Such a shift, if present, may indicate a nuclear regulatory function under pathological conditions. Metaplastic cells exhibited elevated KiSS-1, PI3K, and AKT expression, distinguishing their role in disease progression. Ultrastructural analyses revealed mitochondrial degeneration with electron-dense accumulations and intranuclear inclusions in eutopic tissues—findings that, to our knowledge, have not been previously reported. Collectively, these findings contribute new insights into the cellular pathology of endometriosis and reinforce the relevance of the PI3K/AKT signaling and KiSS-1 expression within its molecular landscape. As far as we know, this is the first comprehensive study to integrate immunofluorescence and ultrastructural comparisons across ectopic, eutopic, and control tissues.

Kisspeptin-expressing neurons, encoded by the KiSS-1 gene, are essential regulators of the hypothalamic—pituitary—gonadal (HPG) axis (De Roux et al., 2003; Beymer et al., 2014). KiSS-1 is also present in the endometrium, where it may influence uterine growth (Cejudo Roman et al., 2012; Leon et al., 2016), and its disruption is linked to gynecologic disorders including endometriosis (Zhang et al., 2023). Prior studies typically combined early (Stage 1–2) and late (Stage 3–4) disease stages without assessing stage-specific cellular localization patterns (İyidoğan et al., 2003; Makri et al., 2012; Roshangar et al., 2013). A key strength of the present study lies in its stage-focused evaluation, which revealed significant variations in KiSS-1 localization across cellular compartments. Earlier reports generally described predominantly cytoplasmic staining and and lower KiSS-1 levels in eutopic tissue from endometriosis patients (Baba et al., 2015; Abdelkareem et al., 2020). However, these prior studies were limited by small sample sizes and lack of rigorous statistical evaluation. More recently, Kleimenova et al. (2024) reported diminished KiSS-1 expression in ectopic lesions, predominantly within glandular regions. These findings differ from ours, which demonstrated notable stromal and occasional nuclear staining. Such discrepancies may be attributed to differences in tissue processing, staging protocols, or analytical sensitivity.

Kisspeptin may exert anti-invasive effects by downregulating stromal-derived factor-1 (SDF-1/CXCL12) and modulating pathways such as PI3K/AKT, which regulate cell survivor and proliferation (Taylor et al., 2014; Chen et al., 2016; Sun et al., 2023; Zhang et al., 2023). The PI3K/AKT pathway is a key survival mechanism enabling ectopic cells to withstand hypoxia and inflammation (Cao et al., 2017; Li et al., 2018; Liu et al., 2019; Madanes et al., 2020). Elevated phosphorylated AKT (pAKT) in ectopic tissues correlate with decreased apoptosis and enhanced survival, possibly stimulated by local estrogen signaling (Garcia-Velasco and Arici, 2003; Beliard et al., 2004; Goumenou et al., 2004; Zhang et al., 2010; Wang et al., 2020; Tian et al., 2023). Our findings of increased AKT activation in both eutopic and ectopic tissues support these observations (Çınar et al., 2009; Kim et al., 2014). Although targeting PI3K/AKT remains a promising therapeutic approach avenue, further research is needed to clarify its role in lesion formation and progression.

The reduction and disruption of glandular structures in ectopic lesions may be attributed to chronic inflammation, excessive stromal fibrosis, and altered extracellular matrix composition. These processes impair glandular differentiation and organization. Moreover, microenvironmental stress conditions—particularly hypoxic and inflammation—may influence PI3K/AKT signaling dynamics by promoting nuclear or perinuclear PI3K localization. Such adaptations could contribute to the heterogeneous immunoreactivity observed across ectopic lesions and further reinforce the pathway’s involvement in disease persistence.

### 4.1 Integration of nuclear KiSS-1 translocation

Emerging evidence indicates that several cytoplasmic proteins can translocate into the nucleus where they exert transcriptional regulatory functions. Zhou et al. (2021) demonstrated that nuclear translocation of GDF15 modulates cancer-related genes, including KiSS-1, through Sp1-dependent promoter activation, ultimately inhibiting cell proliferation and invasion. Similarly, Mueller et al. (2011) showed that nuclear KiSS-1 can act as a transcriptional co-regulator involved in tumor suppression. Consistent with these findings, our study detected nuclear KiSS-1 in both eutopic and ectopic endometrial stromal cells, particularly in stage III eutopic tissue.

While paraffin embedding is routinely used for histology, our findings confirm that JB-4 embedding provides superior cytological resolution for identifying subcellular protein localization. The glycol methacrylate-based resin allows ultrathin (∼1 μm) sections with minimal artifacts, enabling clearer visualization of nuclear and cytoplasmic structures. In contrast, conventional paraffin sections (3–4 µm) may fail to reveal nuclear KiSS-1 due to their lower optimal resolution. Thus, JB-4 embedding offered a technical advantage in confirming nuclear KiSS-1 localization, despite being less commonly used than paraffin or cryosectioning (Bulut, 1996; Mulisch and Welsch, 2015). Future methodological improvements, including optimized antigen retrieval, may further enhance immunostaining compatibility.

The presence of KiSS-1 within the nucleus suggests that it may translocate under pathological stress conditions—such as hypoxia and chronic inflammation—shifting from its traditional cytoplasmic metastasis-suppressor role to a transcription-modulating function. These observations suggest that nuclear KiSS-1 may participate in integrating stress signals into gene-regulatory programs; further studies are required to confirm the functional relevance of this translocation in endometriotic tissues. Understanding this regulatory could also shed light on how endometrial cells evade apoptosis yet avoid malignant transformation.

Nuclear KiSS-1 frequently coincided with Type-1 intranuclear inclusions, mitochondrial degeneration, and stromal fibrosis in our samples. These features support the interpretation that nuclear KiSS-1 participates in a broader stress-adaptive response. Ultrastructural findings—including nuclear indentations and inclusion bodies—are consistent with previously described markers of nuclear stress (Schwertheim et al., 2020; Allavena et al., 2015). Moreover, recent work on nucleophagy indicates that selective degradation of nuclear components contributes to maintaining nuclear homeostasis under stress (Li and Nakatogawa, 2022; Ji et al., 2025). In this context, nuclear KiSS-1 may function as a co-regulator of stress-response genes, aiding cellular adaptation in chronically inflamed or hypoxic endometrial environments. Our ultrastructural observations—mitochondrial abnormalities and Type-1 intranuclear inclusions—provide a morphological context for PI3K/AKT activation and nuclear KiSS-1 translocation. The presence of KiSS-1 within the nucleus suggests that it may translocate under pathological stress conditions, such as hypoxia and chronic inflammation, shifting from its traditional cytoplasmic metastasis-suppressor role to a transcription-modulating function. Mitochondrial stress may trigger AKT phosphorylation, promoting cell survival under hypoxic and inflammatory conditions, while nuclear inclusions could facilitate KiSS-1 recruitment to the nucleus, enabling transcriptional regulation of anti-proliferative and anti-invasive genes (Mattam et al., 2021). Supporting our observations, KiSS-1 has been reported to regulate proliferation and apoptosis via PI3K/AKT/ERK signaling in ovarian granulosa cells in polycystic ovary syndrome (Sun et al., 2023). By analogy, altered KiSS-1 localization in eutopic and ectopic endometrial cells may similarly modulate PI3K/AKT-driven survival pathways, influencing stress adaptation, mitochondrial dynamics, and nuclear stress responses (Beddows et al., 2024; Sohel et al., 2025). This integrative perspective links morphological changes directly to molecular adaptations that sustain lesion survival and restrict excessive proliferation, strengthening the mechanistic interpretation of our findings.

The “eutopic endometrium determinism” theory proposes that intrinsic abnormalities within eutopic endometrium—beyond retrograde menstruation—may drive the development of endometriosis (Gao et al., 2019; Mikhaleva et al., 2021). Our study exhibited significant morphological, morphometric, and ultrastructural alterations in both eutopic and ectopic endometrial tissues, particularly during the proliferative phase. JB-4 embedding revealed chronic stress markers such as complex atypical hyperplasia, stromal reduction, lymphocyte aggregations, tubal metaplasia, and Russell bodies, potentially linked to PI3K/AKT activation. It is important to distinguish tubal metaplasia—marked by ciliated and secretory epithelial features resembling fallopian tube epithelium—from Müllerian metaplasia, which involves transformation between different Müllerian-derived epithelial types. Clarifying this distinction may help determine whether the observed alterations represent adaptive responses or intrinsic developmental abnormalities. Although ultrastructural and cytological findings could suggest early stress-adaptive or even premalignant transformations, these interpretations should remain cautious. Morphology and morphometry alone cannot establish carcinogenic potential. Future research incorporating molecular analyses—such as p53 status, autophagy-related markers (LC3, Beclin-1), and cell-cycle regulators—is required to validate these hypotheses indicate a preneoplastic trajectory. In advanced stages, such metaplastic changes may mimic precancerous conditions, complicating the histopathological interpretation of endometriosis. Furthermore, altered kisspeptin expression hints at role in immune modulation and inflammatory modulation, warranting further investigation.

Transmission electron microscopy (TEM) revealed striking ultrastructural abnormalities in eutopic tissues, even more pronounced than in ectopic lesions. Key morphological changes included microplicae on the apical surface, deep nuclear indentations, intranuclear inclusion bodies, and marked mitochondrial degeneration—all features indicative of cellular stress and impaired function. Since microvilli and microplicae are functionally associated with implantation and embryo-endometrial interactions, any disruption of these structures could compromise trophoblast attachment and normal decidualization (Elgamal et al., 2016). Both light and electron microscopy also revealed the presence of decidua-like cells, macrophages, and spindle-shaped fibroblasts within the endometriotic tissue, contributing to the immune-modulated and fibrotic microenvironment characteristic of this disease. Morphometric analyses corroborated the ultrastructural findings, demonstrating reduced stromal cell density and increased collagen fiber deposition in ectopic lesions compared with controls. The proliferation of irregularly distributed collagen fibers, clearly visualized in both light and electron microscopy, aligns with previous studies documenting enhanced fibroblastic activity and extracellular matrix accumulation in endometriotic stroma (Dilsiz et al., 2013). Despite minor cytological changes were observed in the control group, epithelial atypia and structural abnormalities were markedly more prominent in endometriosis tissues. These findings contribute to growing evidence that eutopic endometrium in endometriosis may exhibit features associated with cytological atypia and hyperplasia, raising concerns about its potential involment in premalignant pathways. Previous studies have proposed a link between atypical endometriosis and the development of ovarian clear cell and endometrioid carcinomas (LaGrenade and Silverberg, 1988; Moll et al., 1990; Munksgaard and Blaakaer, 2012; Li et al., 2023). Larger cohort studies have reinforced these observations, reporting higher rates of atypia in cancer-associated endometriosis compared to benign cases (Fukunaga et al., 1997; Ogawa et al., 2000; Prefumo et al., 2002). Although a direct causal relationship remains uncertain, shared molecular characteristics suggest a possible pathogenic continuum. Genomic and longitudinal studies are needed to clarify whether atypical endometriosis truly represents a premalignant state.

Ultrastructural analysis revealed clear deviations from normal stromal cell morphology described by Dockery et al. (1990) and Cornillie et al. (1985), including increased nuclear heterochromatin and prominent intranuclear inclusions. Our observations suggest the presence of Type-1 intranuclear inclusion cysts in stromal, glandular, and luminal epithelial nuclei; to our knowledge, such findings have not been widely reported. Such inclusions may reflect nuclear sequestration or autophagic responses triggered by chronic cellular stress, consistent with the interpretations of Schwertheim et al. (2020). Their autophagy-related content supports the possibility that nuclear quality-control mechanisms are activated in endometriotic tissue. Previous work described that autophagy can protective or deleterious depending on context (Allavena et al., 2015; Yang et al., 2017; Sui et al., 2018; Ding et al., 2021; Peng et al., 2022). Taken together, the inclusions observed in our samples may therefore represent an adaptive nuclear response rather than degeneration.

Ectopic endometrial cells display neoplastic-like features such as abnormal proliferation, migration, and resistance to apoptosis (Matsuzaki and Darcha, 2015), which are closely linked to mitochondrial function. Mitochondria play a crucial role in regulating apoptosis, metabolism, and ROS detoxification—processes critical in endometriosis pathophysiology. The hypoxic and inflammatory peritoneal environment where exfoliated endometrial cells implant further exacerbates mitochondrial stress (Ren et al., 2022). Although mitochondrial dysfunction is well-documented in gynecologic diseases (Hu et al., 2017; Signorile et al., 2019; Ye et al., 2023), specific alterations in ectopic endometrial stromal cells remain underexplored. Recent studies report elevated ROS and ATP production in ectopic tissues, alongside upregulated SOD2 expression, suggesting a role in oxidative stress management and cellular survival (Chen et al., 2019). SOD2 not only neutralizes ROS but also promotes proliferation and migration. Mitochondrial morphology is known to adapt dynamically to stress; hypoxia induces elongation to preserve energy, while fragmentation occurs with impaired OXPHOS (Jendrach et al., 2008; Ryu et al., 2020). In contrast, our ultrastructural findings revealed significant mitochondrial abnormalities—including vacuolization, cristae disruption, and dense deposits—in both stromal and epithelial cells of ectopic and eutopic tissues. These alterations suggest impaired mitochondrial dynamics, reduced ATP synthesis, and increased apoptotic susceptibility. Disruption of the fission/fusion balance may compromise mitochondrial quality control. We propose that chronic oxidative stress in the endometriotic microenvironment forces mitochondria to maintain energy production, potentially driving lesion survival. The upregulation of DRP1 under hypoxia (Kobashigawa et al., 2011) and the downregulation of mitochondrial dynamics-related genes (Ye et al., 2023) may reflect an adaptive mitochondrial reprogramming during ectopic establishment. However, additional research is required to fully elucidate these mechanisms.

A key limitation of our study is the modest sample size, which reduce the generalizability of the ultrastructural findings. Additionally, the absence of functional assays—such as Western blotting or peptide-blocking experiments—restricts confirmation of protein expression and subcellular localization observed *via* immunofluorescence and TEM. However, the implementation of established positive and negative controls, combined with the reproducibility of staining patterns consistent with prior literature, reduces the likelihood that our observations represent technical artifacts. Larger cohorts and integrated molecular analyses are warranted to validate these conclusions.

In conclusion, our study highlighted key biological and ultrastructural distinctions among eutopic, ectopic, and normal endometrium. The findings highlight the dual role of KiSS-1—both cytoplasmic and nuclear—in maintaining endometrial homeostasis and restraining aberrant proliferation. The interplay between KiSS-1 nuclear translocation, PI3K/AKT signaling, and mitochondrial stress may represent a pivotal axis in endometriosis pathophysiology. From the broader cancer biology perspective, KiSS-1 functions as a metastasis suppressor through multiple complementary mechanisms. In various malignancies, KiSS-1/KiSS-1R signaling inhibits metastatic progression by downregulating NF-κB and MMP-9, suppressing epithelial-mesenchymal transition (EMT), reducing CXCR4-mediated chemotactic migration, and activating MAPK and PKC pathways to enforce anti-proliferative signals (Lee and Welch, 1997; Ohtaki et al., 2001; Yan et al., 2001; Ringel et al., 2002; Gründker et al., 2015; Song and Zhao, 2015). The secreted kisspeptin peptides act in a paracrine or autocrine fashion, influencing surrounding stromal or immune cells even when tumor cells themselves lack KiSS-1R, generating growth-inhibitory microenvironments (Nash and Welch, 2006; Beck and Welch, 2010). Mechanistically, KiSS-1R is a Gq/11-coupled receptor that triggers phospholipase C activation, IP3/DAG-mediated intracellular Ca^2+^ release, and downstream MAPK (ERK1/2, p38) signaling, leading to inhibition of cell motility, invasiveness, and angiogenesis (Kotani et al., 2001; Muir et al., 2001). KiSS-1 also interferes with CXCR4/SDF-1 signaling, attenuating chemotaxis and metastasis potential (Sousa and Espreafico, 2008), and engages PP2A-mediated Akt dephosphorylation, promoting apoptosis in susceptible cells (Navenot et al., 2009; Pampillo et al., 2009). Collectively, these mechanisms highlight a dual functional role of KiSS-1: intracellular/nuclear regulation, as observed in endometriotic cells, and extracellular modulation of the microenvironment, reflecting a conserved stress-response and anti-invasive function. This integrated mechanistic view provides a strong rationale for exploring KiSS-1 analogues or agonists to therapeutically modulate lesion progression or invasive behavior in endometriosis and other pathologies sharing metastatic-like features (Matsui and Asami, 2014; Blake et al., 2017). Further multi-omic analyses are needed to define nuclear KiSS-1–regulated gene networks and explore its potential as a biomarker or therapeutic target. Given the distinct nuclear and cytoplasmic localization patterns of KiSS-1 in eutopic and ectopic endometrium, future studies could explore its potential as a diagnostic or prognostic biomarker for endometriosis progression. Integration with PI3K/AKT pathway activity and mitochondrial stress markers may enable the development of multi-parametric panels to stratify patients based on lesion invasiveness or recurrence risk, potentially informing personalized therapeutic strategies.

## Data Availability

The datasets presented in this study can be found in online repositories. The names of the repository/repositories and accession number(s) can be found in the article/Supplementary Material.

## Supplementary material

SUPPLEMENTARY FIGURE S1 Expression levels of KISS-1, PI3K, and AKT-1 proteins in prostate tissue samples from positive controls. Representative images are shown for each group. Protein expression was detected using immunofluorescence, and differences between groups were evaluated. Positive controls show expected expression patterns for each target protein, whereas negative control demonstrate minimal or no staining, confirming antibody specificity. Since all antibodies are mouse monoclonal, only a single image was used as the negative control, which was shared for all three proteins. Scale bars = 10 µm.

SUPPLEMENTARY FIGURE S2 Morphological findings of control endometrial sections obtained with the JB4 technique. LE: Luminal epithelium, GE: Glandular epithelium, Ciliate cells (Brown arrow), secretory granules (purple arrow), Atypical cells (green asterisk), Mitotic figures (yellow arrows), Stromal cell (yellow arrowhead). Scale bars = 10 µm.

SUPPLEMENTARY FIGURE S3 Immunofluorescence localizations of KiSS-1 protein in endometrial tissues of eutopic and control groups. GE: Glandular epithelium, LE: Luminal epithelium, Atypical cells (purple arrow), perinuclear staining (White arrow). Scale bars = 10 µm.

SUPPLEMENTARY FIGURE S4 Immunofluorescence localizations of PI3K protein in endometrial tissues of eutopic and control groups. GE: Glandular epithelium, LE: Luminal epithelium. Scale bars = 10 µm.

SUPPLEMENTARY FIGURE S5 Immunofluorescence localizations of AKT-1 protein in endometrial tissues of ectopic, eutopic and control group patients. LE: Luminal epithelium, ESc: Endometrial stromal cells, Atypical cells (White arrow). Scale bars = 10 µm.
