# Supplementary figures and images for: Evidence for KISS-1 nuclear translocation and PI3K/AKT signaling in the ultrastructurally and morphometrically analyzed human endometriosis
Source: Front Cell Dev Biol. 2026 Jan 6;13:1625031. doi: 10.3389/fcell.2025.1625031 (PMC12816297; doi:10.3389/fcell.2025.1625031)

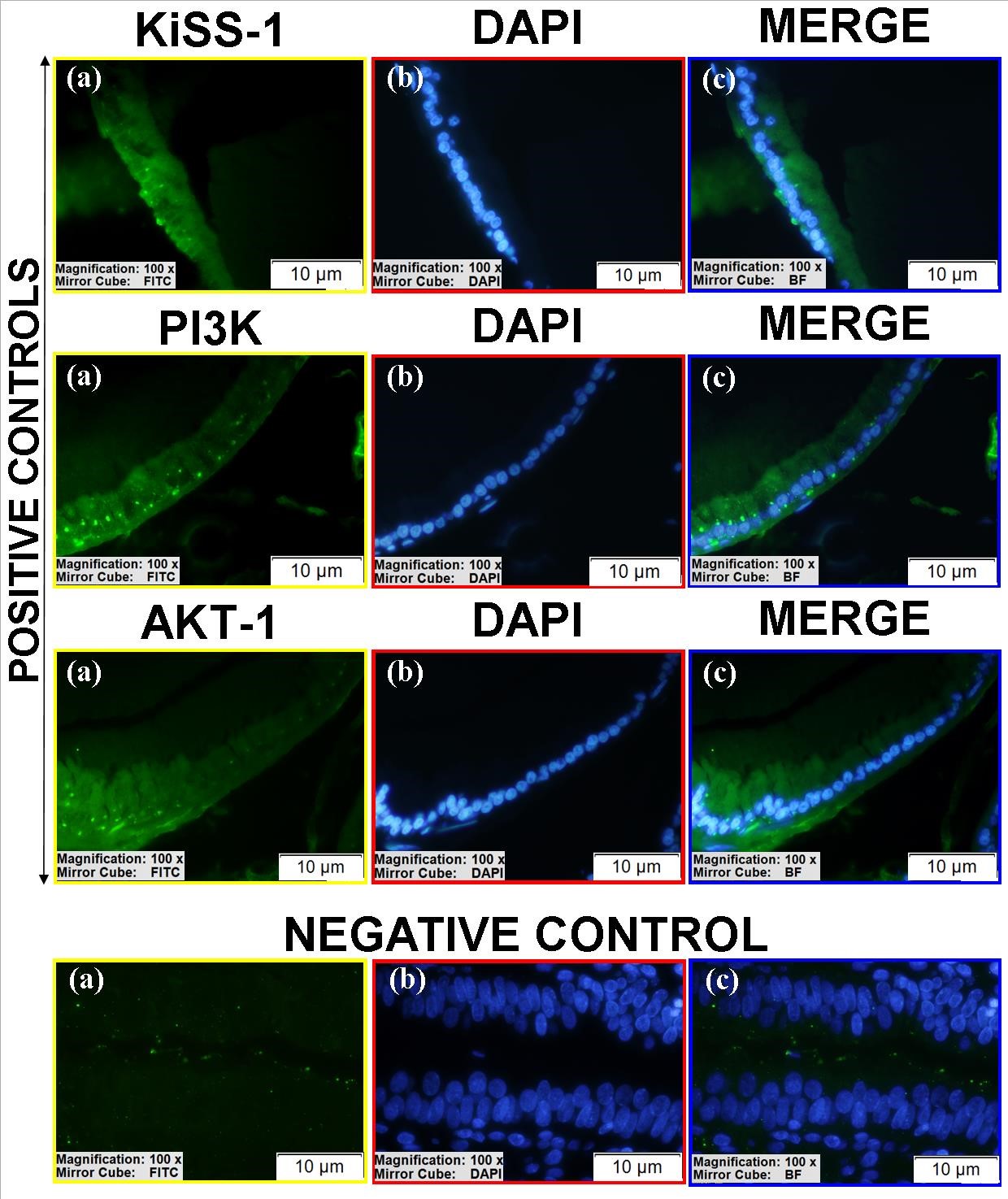

Supplement: Supplementary file 1 [file DataSheet1.zip › Image 1.JPEG]

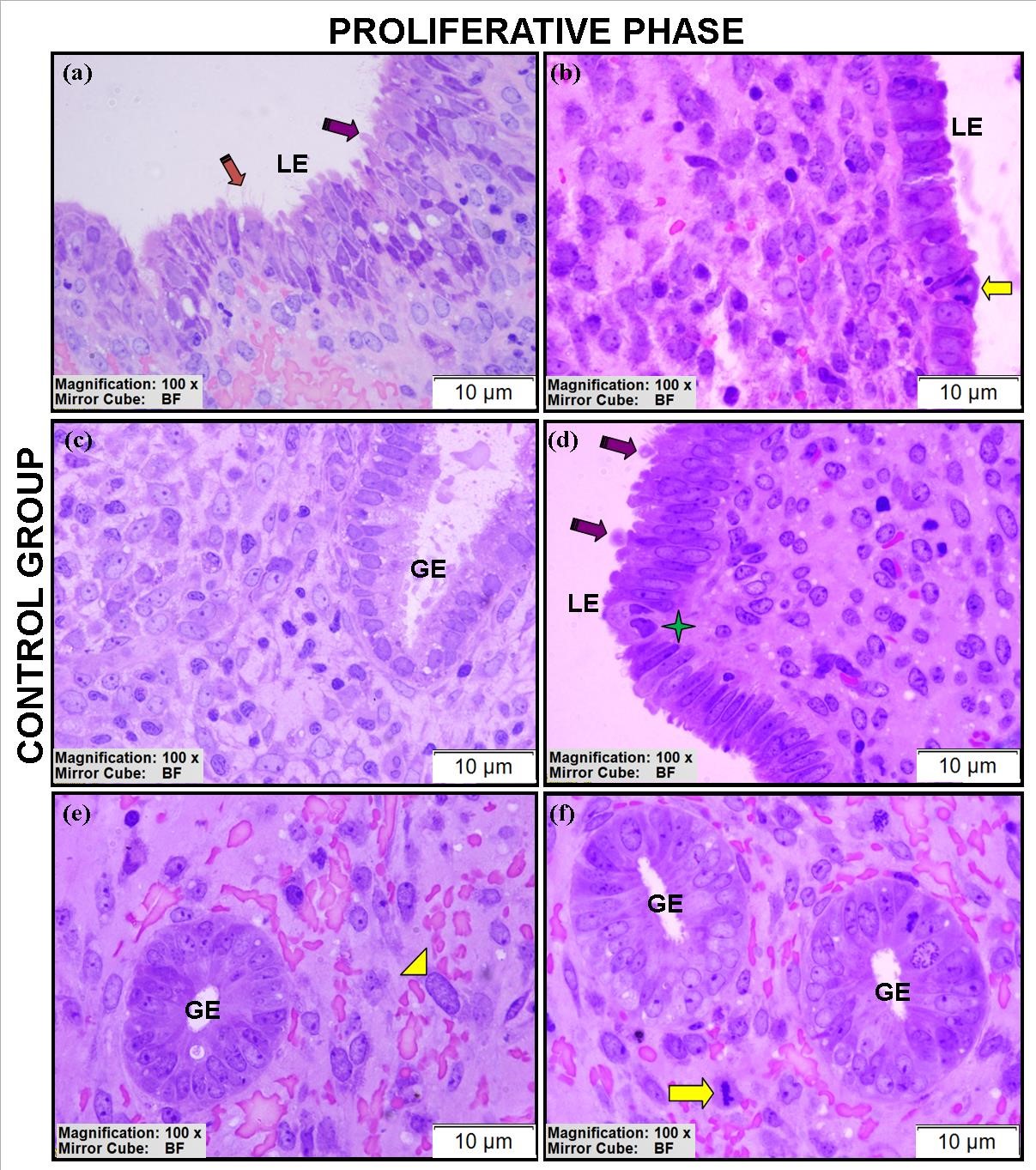

Supplement: Supplementary file 1 [file DataSheet1.zip › Image 2.JPEG]

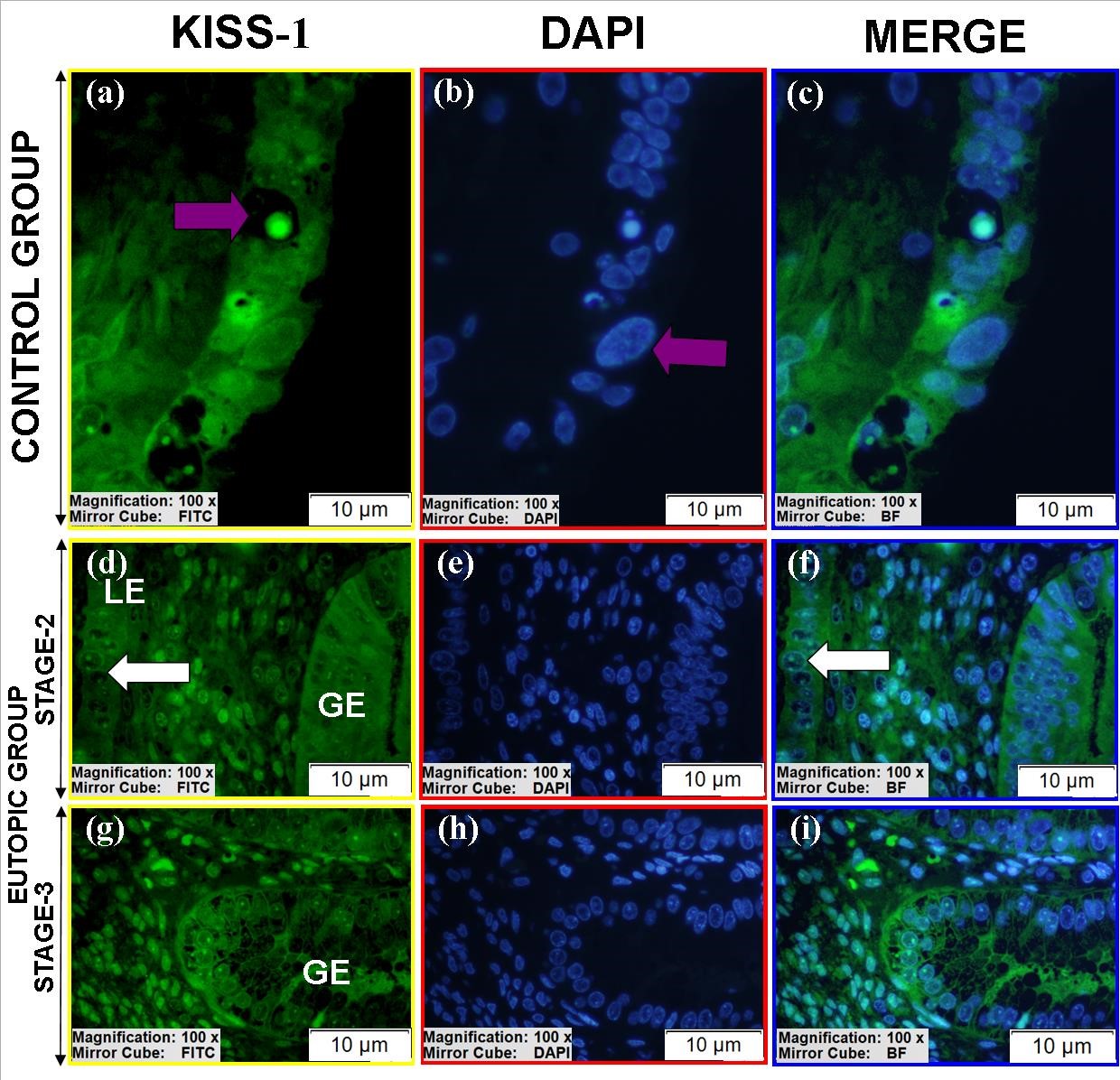

Supplement: Supplementary file 1 [file DataSheet1.zip › Image 3.JPEG]

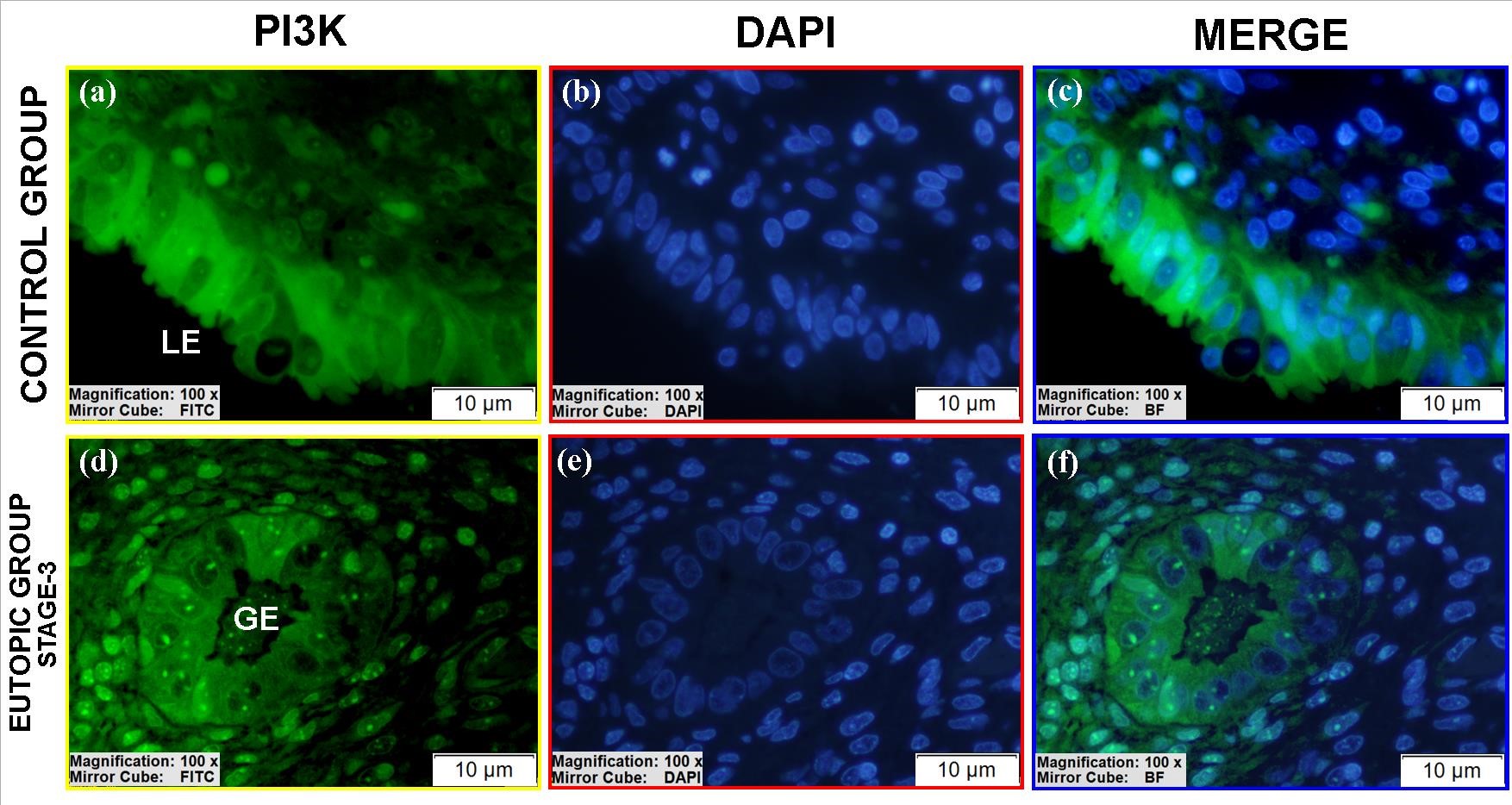

Supplement: Supplementary file 1 [file DataSheet1.zip › Image 4.JPEG]

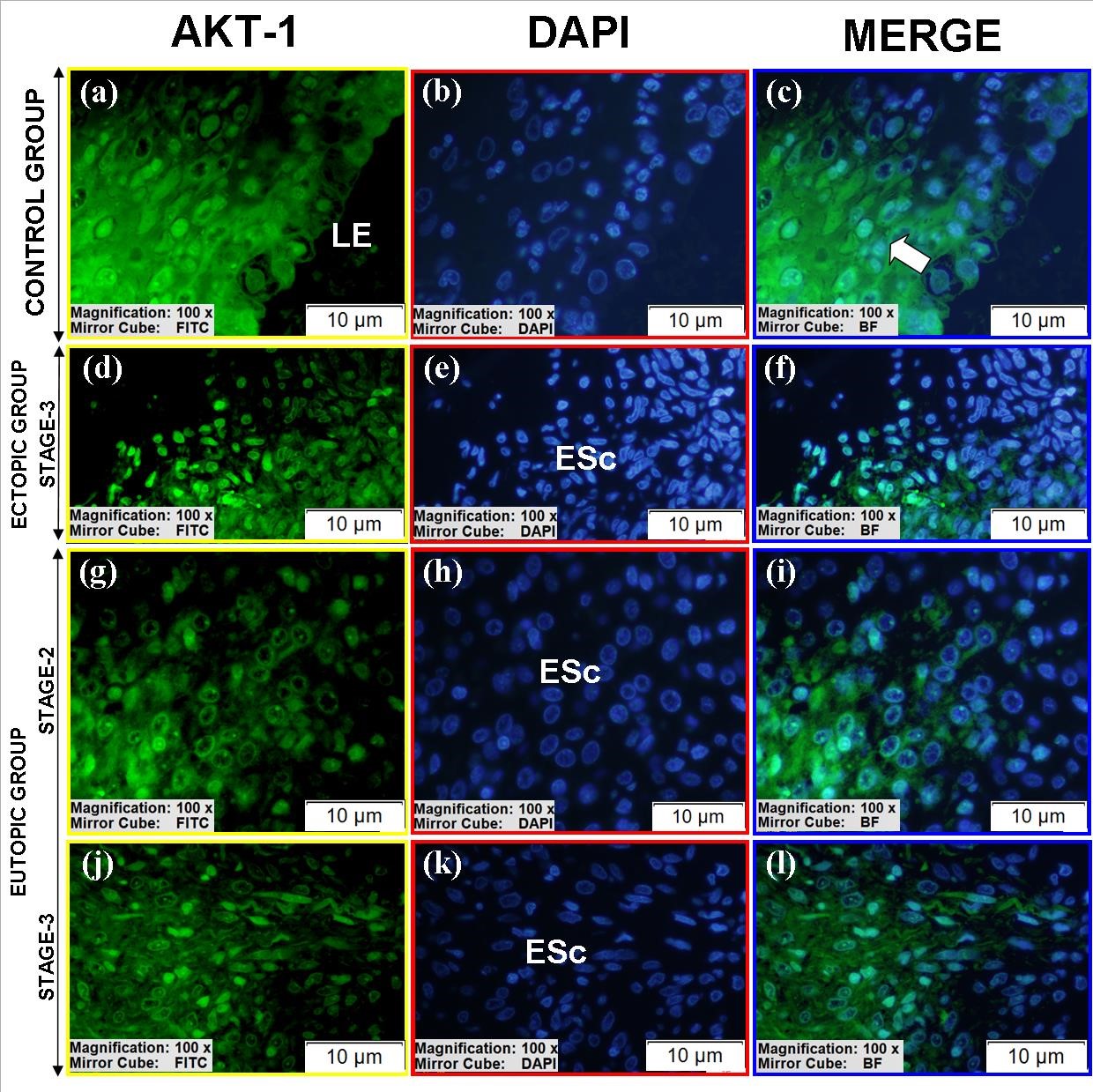

Supplement: Supplementary file 1 [file DataSheet1.zip › Image 5.JPEG]
